# Evaluation of the Control Efficacy of Bt Maize Expressing Cry1Ab and Vip3Aa Proteins Against *Agrotis ypsilon* (Rottemberg)

**DOI:** 10.3390/insects16020119

**Published:** 2025-01-26

**Authors:** Wenhui Wang, Yuting He, Huan Yu, Xianming Yang, Kongming Wu

**Affiliations:** 1 State Key Laboratory for Biology of Plant Diseases and Insect Pests, Institute of Plant Protection, Chinese Academy of Agricultural Sciences, Beijing 100193, China; w975480209@163.com (W.W.); 17855599733@163.com (Y.H.); 18511627822@163.com (H.Y.); zqbxming@163.com (X.Y.); 2 Institute of Insect Sciences, College of Agriculture and Biotechnology, Zhejiang University, Hangzhou 310058, China; 3 Department of Entomology, China Agricultural University, Beijing 100193, China

**Keywords:** *Agrotis ypsilon*, Bt maize, susceptibility, preference

## Abstract

Bt maize is the main means to control many lepidopteran pests in the world, but its control efficacy against *Agrotis ypsilon* (Rottemberg), an important insect pest of maize seedlings, remains unclear until now. The interaction between the insect and Bt transgenic maize events (DBN9936 (expressing Cry1Ab), DBN9501 (expressing Vip3Aa), and DBN3601T (expressing Cry1Ab and Vip3Aa)) was investigated using bioassay and insect behavioral tests. The results show that the Cry1Ab contents in different tissues of DBN9936 were 47.78–82.60 μg·g^−1^, and the Vip3Aa contents in DBN9501 were 15.29–27.78 μg·g^−1^. The contents of Cry1Ab and Vip3Aa in DBN3601T were 32.08–79.08 and 10.16–17.52 μg·g^−1^, respectively. There was no significant difference in total Bt protein content between the leaves and stems; however, that the content in both was significantly higher than that in the roots. The larvae were most sensitive to the Vip3Aa protein, and the corrected mortalities of larvae feeding on DBN9501 and DBN3601T were greater than 89.65% at the seedling stage, significantly higher than those feeding on DBN9936 (16.46–76.13%). The corrected mortalities of the third to the fifth instar larvae feeding on Bt maize root were as follows: DBN3601T (54.00–96.60%) > DBN9501 (24.67–70.88%) > DBN9936 (6.67–53.31%). The results of behavioral tests for Bt/non-Bt maize plant selection indicated that the larvae mainly fed on non-Bt maize while showing antifeedant behavior toward Bt maize, and the moth preferred to lay eggs on undamaged or slightly damaged Bt maize. It is concluded that DBN3601T maize has a strong control efficacy for *A. ypsilon*, which can play an important role in building an integrated pest management strategy for the insect.

## Simple Summary

*Agrotis ypsilon* (Rottemberg) is a worldwide major pest of maize seedlings. Currently, Bt maize is the main means of controlling lepidopteran pests, but its control efficacy against *A. ypsilon* is unclear. We studied the toxicity of Bt proteins expressed by DBN9936 (expressing Cry1Ab), DBN9501 (expressing Vip3Aa), and DBN3601T (expressing Cry1Ab and Vip3Aa) against *A. ypsilon*. The bioassay showed that Vip3Aa has the most toxicity and that DBN3601T maize can effectively control the damage of the pest to maize plants. The results of behavioral tests indicated that the insect larvae exhibited antifeedant behavior toward Bt maize, while the moths demonstrated a preference for laying eggs on undamaged or minimally damaged Bt maize plants. This study provided a theoretical basis for building an integrated pest management system for *A. ypsilon* based on Bt maize planting.

## 1. Introduction

*Agrotis ypsilon* (Rottemberg) (Lepidoptera: Noctuidae) is a global underground pest [1], widely distributed in Europe, North America, Asia, Oceania, and other regions, causing serious damage to agriculture and forestry [2]. *A. ypsilon* moths partake in long-distance migration, and the pattern of northward migration in spring and back migration in autumn has intensified the occurrence of harm in Europe, Asia, and North America [3,4,5,6].

The larvae of *A. ypsilon* can damage the seedlings of almost all vegetables and important grains, especially maize, the world’s main food crop [2]. The maize damage can be divided into two stages: firstly, lower-instar larvae feed on the leaves and young tissues of the maize seedling stage; in the second stage, higher-instar larvae drill into the soil surface and damage the roots and stems, resulting in the absence of seedlings and ridges [7,8].

As with other widespread pests, it is difficult to control *A. ypsilon* using a single control measure [1]. At present, the control of *A. ypsilon* mainly depends on chemical pesticides [9], but chemical control is more efficient for low-instar larvae than for high-instar larvae moving underground. However, spraying pesticides on soil can easily pollute soil and water [10], and the long-term use of chemical pesticides leads to the resistance of *A. ypsilon* to organophosphorus, carbamates, and pyrethroid insecticides [11]. Physical control methods are not very efficient and are difficult to apply over large areas as well. As a relatively environmentally friendly technology, biological control has long been demonstrated to be effective, but at a relatively slow pace [12]. In actual applications, challenges such as the immature feeding and release technology for natural enemy insects, the high cost of biopesticides, and their unstable effects also exist. Therefore, it is of great significance to seek green, efficient, and low-toxicity control methods to prevent and control *A. ypsilon*.

Crops expressing specific insecticidal proteins, such as *Bacillus thuringiensis* (Bt), have achieved significant success in controlling target pests. Since the first genetically modified (GM) crops were planted in 1996, GM insect-resistant crops have been planted in 29 countries worldwide, with the global area of GM crops reaching 206 million hectares in 2021 [13]. The commercially available GM insect-resistant crops mainly include cotton, maize, and soybeans. The control of target pests by GM insect-resistant crops is significant, with Bt maize effectively reducing the damage caused by *Spodoptera frugiperda* (fall armyworm) [14], *Helicoverpa armigera* (cotton bollworm) [15], *Ostrinia furnacalis* (corn earworm) [16,17], and some Coleoptera pests. Unlike conventional broad-spectrum insecticides, Bt proteins have selective toxicity to pests; for example, Cry1Ab-expressing Bt maize primarily targets the corn earworm but shows poor control of *Mythimna separata* [18]. Vip3Aa-expressing Bt maize is more toxic to the fall armyworm than the cotton bollworm [19,20]. Only a few studies have assessed the susceptibility of *A. ypsilon* to Bt proteins, such as those demonstrating that the Vip3Aa toxin is more toxic than Cry1Ab to *A. ypsilon* [21], Cry2Ab is more toxic to *A. ypsilon* than Cry1Ac [22], and Cry1F has some insecticidal effects on *A. ypsilon*, while Cry3 proteins have no significant effects on them [23].

China is accelerating the commercialization of Bt maize, with 12 Bt maize varieties having obtained biosafety certificates as of 2024 [24]. Research has shown that Bt maize has high control efficacy for the fall armyworm [25], *M. separata* [26], the corn earworm [27], and the cotton bollworm [28]. However, the control efficacy of Bt maize for *A. ypsilon* is still unclear. In this study, we investigated the toxicity of Bt maize to *A. ypsilon* and its effects on their feeding and oviposition preferences. The results provide scientific evidence for the subsequent commercial cultivation of Bt maize and integrated management of *A. ypsilon*.

## 2. Materials and Methods

### 2.1. Materials

The *A. ypsilon* strain was provided by the Langfang Experimental Station, Chinese Academy of Agricultural Sciences in Hebei province. The adult feed consisted of 10% sugar water, and eggs were collected every day. The larvae were raised on an artificial diet [29], and different instar larvae were raised for the experiment. All the larvae and moths were put in a climate-controlled chamber with a temperature of 26 ± 1 °C, relative humidity (RH) of 70 ± 10%, and a photoperiod of 16 h:8 h (light/dark).

The seeds in the test were provided by the Dabeinong (DBN) Group Beijing, China. DBN9936 (expressing the Cry1Ab protein), DBN9501 (expressing the Vip3Aa protein) [30], DBN3601T (expressing the Cry1Ab and Vip3Aa proteins) [31], and the non-Bt maize Zhengdan 958 were all planted in a greenhouse at 26 °C. When the maize plant had grown to the 4-leaf stage, the leaves, stems, and roots were cut; part of the maize was freeze-dried, ground into powder, and then stored in a freezer at −80 °C; and the other part was used for a bioassay.

### 2.2. ELISA Determination of the Contents of Bt Protein in Different Tissues of Bt Maize Seedlings

Enzyme-linked immunosorbent assay (ELISA) was used to determine the contents of the Bt protein in freeze-dried maize in different tissues. The Cry1Ab protein was determined using the Cry1Ab/Cry1Ac Quantiplate Kit (Envirologix, Portland, ME, USA), the Vip3Aa protein was determined using the Vip3A Quantiplate Kit (YouLong Biotech, Shanghai, China), and all operations were performed according to the instructions.

### 2.3. Determination of Toxicity of Bt Protein Expressed by Bt Maize to Larvae

According to the content of the Bt protein in the freeze-dried maize leaf powder, the powder was diluted with an artificial diet to various concentrations. The diluted concentrations of Cry1Ab expressed in DBN9936 were 0.4131, 0.8263, 1.6526, 3.3052, and 6.6104 μg·g^−1^. The diluted concentrations of Vip3Aa expressed in DBN9501 were 0.1236, 0.2472, 0.4944, 0.9888, and 1.9776 μg·g^−1^. The diluted concentrations of Cry1Ab + Vip3Aa expressed in DBN3601T were 0.4830, 0.9660, 1.9320, 3.8640, and 7.7280 μg·g^−1^. A corresponding amount of Zhengdan 958 maize leaf lyophilized powder was mixed into the artificial diet as a control [32].

The diluted toxin diets (0.5 g) were added along with one neonate *A. ypsilon* to each well of 24-well plates in a completely randomized fashion, with 4 replicates per concentration and 24 larvae per replicate at each concentration. The plates were then placed in a chamber. Depending on the freshness of the diet and the amount consumed, the diet was replaced or added during the 14-day assay. After 14 days, we counted any dead larvae that did not crawl normally when touched with a brush. We then calculated the mortality and corrected mortality.

### 2.4. Determination of Mortality of Larvae Feeding on Bt Maize

Maize seedlings at the 4-leaf stage were cut and inserted into glass tubes containing 2% agar, and the 1st to 3rd instar larvae were put on the leaves, with 50 individuals in each group and 3 replicates. The maize stem base and root were placed in a disposable Petri dish, and the 3rd to 5th instar larvae were put on the maize stem base and root, with 50 individuals in each group and 3 replicates. The maize tissue was changed every day, and the survival condition of the larvae was investigated until death. The mortality rate, corrected mortality rate, and survival days of the larvae were recorded every day.

### 2.5. Determination of Feeding Preference of Larvae for Bt Maize and Non-Bt Maize

Maize seedlings at the 4–6 leaf stage were planted for the experiment. The feeding preference of the larvae for Bt maize and non-Bt maize was determined using the Han et al. (2015) method [33]. Eight 3 cm leaf segments of each maize sample were randomly cut and placed in a disposable Petri dish (diameter: 15 cm) containing 1 cm thick agar at equal intervals. Forty newly hatched larvae, starved for 2 h, were put in the center of the dish, covered with disposable plastic wrap, and then covered with black cloth. The dishes were transferred to the chamber, with 15 replicates. The number of larvae feeding on the leaves of the 4 maize varieties was observed and recorded after 5 h, and then, the feeding selection rate was calculated.

### 2.6. Determination of Ovipositional Preference of Moth for Bt Maize and Non-Bt Maize

The ovipositional preference of the moth was measured in the cage. Two conditions were established: the first condition, where (1) neither Bt maize nor non-Bt maize was damaged. Seedlings of DBN9936, DBN9501, DBN3601T, and non-Bt maize, with the same planting density, about 20 cm high, and with a similar leaf area, were randomly placed in a nylon mesh cage (40cm × 40cm × 40cm, 120 mesh). Fifteen pairs of adult moths (female/male = 1:1) that newly emerged on the same day were placed in the cage and randomly matched. A cotton ball filled with 10% sucrose water was hung at the top in the middle of the cage to supplement nutrition. Eggs on the whole plants were collected and counted every day until the moths no longer laid eggs. The experiment was carried out in a greenhouse (26 ± 1 °C) with 5 replicates. The second condition was (2) where both Bt maize and non-Bt maize were damaged. When the maize plants had grown to the 4-leaf stage, 10 newly hatched larvae were placed on Bt maize and non-Bt maize to damage the leaves and cause holes. After 2 days, maize with obvious damage was used for the ovipositional preference experiment. The subsequent experimental operation was the same as for (1). Eggs on the whole plants were collected and counted every day until the moths no longer laid eggs. The experiment was carried out in a greenhouse (26 ± 1 °C) with 3 replicates.

### 2.7. Statistical Analysis

The LC_50_ values and 95% fiducial limits were calculated by probit analysis, and the Slope ± SE, *χ^2^*, *df*, and *p* values were calculated. Two-sample *t*-tests and one-way analysis of variance (ANOVA) were used to compare the differences in the contents of the insecticidal protein expressed by different Bt maize and the differences in the mortality and selectivity of *A. ypsilon* on Bt maize (*p* < 0.05). All the data were analyzed using SPSS 23.0 software.


Mortality rate (%) = (Number of dead larvae after treatment)/(Number of total larvae) × 100.

Corrected mortality (%) = [(Mortality rate of larvae on Bt maize − Mortality rate of larvae on non-Bt maize)/(1 − Mortality rate on non-Bt maize)] × 100.

## 3. Results

### 3.1. Expression of Bt Protein in Different Tissues of Bt Maize Seedlings

**Figure 1:**
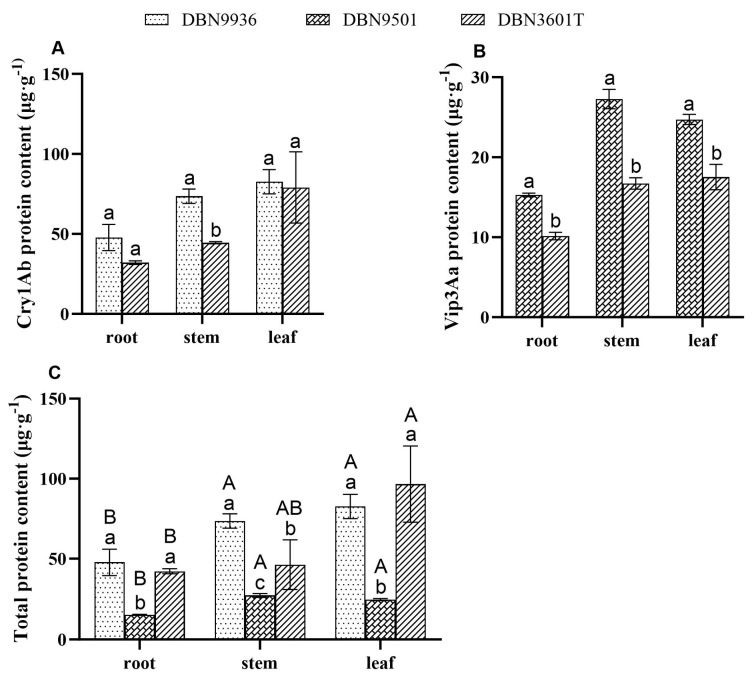
The contents of the Bt protein in the roots, stems, and leaves of different Bt maize seedlings. (**A**) The contents of Cry1Ab in different Bt maize seedlings in the tissue; (**B**) The contents of Vip3Aa in different Bt maize seedlings in different tissues; (**C**) The total Bt protein in different Bt maize seedlings in different tissues. Different lowercase letters indicate significant differences in Bt protein content in the same tissue, and different capital letters indicate that the content of Bt protein in different tissues is significantly different (two-sample *t*-test and one-way ANOVA, *p* < 0.05). μg·g^−1^ means μg of Bt protein per gram of dry weight.

The insecticidal protein contents in different tissues of Bt maize are shown in Figure 1. The Bt proteins were expressed in the roots, stems, and leaves of DBN9936, DBN9501, and DBN3601T. The Cry1Ab contents in the roots, stems, and leaves of DBN9936 were 47.78, 73.55, and 82.60 μg·g^−1^, respectively (Figure 1A). The contents of Vip3Aa in the roots, stems, and leaves of DBN9501 were 15.29, 27.78, and 24.72 μg·g^−1^, respectively (Figure 1B). The Cry1Ab contents in the roots, stems, and leaves of DBN3601T were 32.08, 44.48, and 79.08 μg·g^−1^, respectively (Figure 1A); the Vip3Aa contents were 10.16, 16.72, and 17.52 μg·g^−1^, respectively (Figure 1B); the total Bt protein contents were 42.24, 61.20, and 96.60 μg·g^−1^, respectively (Figure 1C).

There were differences in the Bt protein content among different Bt maize varieties for the same tissue. The Cry1Ab content in the stems of DBN3601T was significantly lower than that in the stems of DBN9936, while there was no significant difference in Cry1Ab content in the roots and leaves (Figure 1A). The Vip3Aa content in the roots, stems, and leaves of DBN3601T was significantly lower than that in those of DBN9501. The total Bt protein content in the roots and leaves of DBN9936 and DBN3601T showed no significant difference but was significantly higher than that in those of DBN9501 (Figure 1B), the total Bt protein content in the stems ranked as follows: DBN9936 > DBN3601T > DBN9501 (Figure 1C). The content of total Bt protein in different tissues was different. There was no significant difference in the total Bt content of leaves and stems, but both were significantly higher than that of the roots. (Figure 1C).

### 3.2. Susceptibilities of A. ypsilon to Bt Protein Expressed in Bt Maize

The LC_50_ of Cry1Ab expressed by DBN9936 was 3.44 μg·g^−1^. The LC_50_ of Vip3Aa expressed by DBN9501 was 0.82 μg·g^−1^, and the LC_50_ of Cry1Ab+Vip3Aa expressed by DBN3601T was 3.39 μg·g^−1^. The susceptibility of larvae to the Bt protein expressed in DBN9936 and DBN3601T was not significantly different between the two but was significantly lower than their susceptibility to the Vip3Aa protein expressed by DBN9501 (Table 1).

### 3.3. Mortality of A. ypsilon Feeding on Bt Maize Seedlings

**Figure 2:**
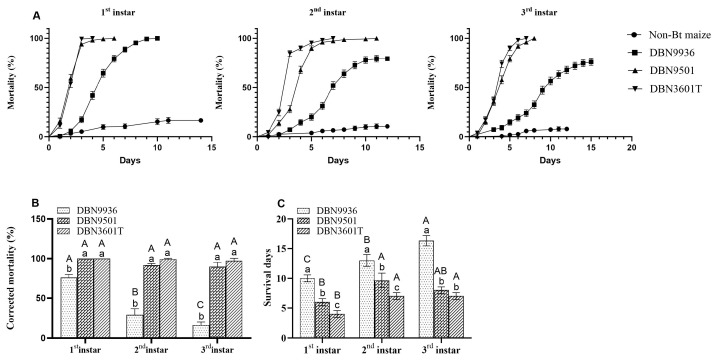
Mortalities of first to third instar larvae on the different maize leaves. (**A**) Mortality curves of different instar larvae on maize leaves; (**B**) The corrected mortality of larvae on different Bt maize leaves (7 d); (**C**) The survival days of different instar larvae on different Bt maize leaves. Different uppercase letters indicate that the corrected mortality and the survival days of different-instar larvae on the same Bt maize were significantly different. Different lowercase letters indicate that the corrected mortality and the survival days of same-instar larvae on different Bt maize were significantly different (one-way ANOVA, *p* < 0.05).

The mortality rate of the first to the third instar larvae feeding on Bt maize leaves gradually increased over the observation days. The mortality rates of larvae of the same instar were different on Bt maize leaves. The mortality curve for the first to the third instar larvae feeding on Bt maize leaves was DBN3601T > DBN9501 > DBN9936 throughout the observation period, while the mortalities on non-Bt maize leaves were all within 20% (Figure 2A).

On day 7, the corrected mortality of the first to the third instar larvae was over 97.21% on DBN3601T, over 89.65% on DBN9501, and between 16.46% and 76.13% on DBN9936. The corrected mortality of same-instar larvae on DBN3601T and DBN9501 leaves was significantly higher than that on DBN9936 leaves. The corrected mortality of DBN9936 maize significantly decreased with increasing instars, but the corrected mortality of DBN3601T and DBN9501 was independent of instar. (Figure 2B). The mortality of larvae at different instars feeding on the same Bt maize leaves also differed. The more advanced the instar stage, the greater the survival duration. (Figure 2C).

**Figure 3:**
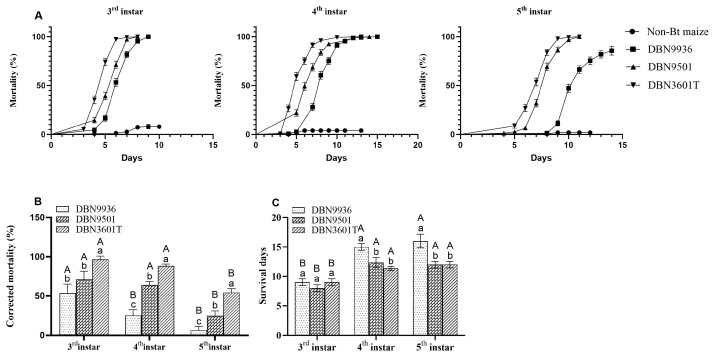
Mortalities of third to fifth instar larvae on the different maize roots. (**A**) Mortality curves of different instar larvae on maize roots; (**B**) The corrected mortality of larvae on different Bt maize roots (7d); (**C**) The survival days of different instar larvae on different Bt maize roots. Different uppercase letters indicate that the corrected mortality and the survival days of different instar larvae on the same Bt maize were significantly different. Different lowercase letters indicate that the corrected mortality and the survival days of same-instar larvae on different Bt maize were significantly different (one-way ANOVA, *p* < 0.05).

The mortality of the larvae in the Bt maize roots gradually increased with observation days. The mortality of the larvae of the same instar was different in Bt maize roots. The mortality curve of larvae from the third to the fifth instars feeding on Bt maize roots was DBN3601T > DBN9501 > DBN9936 throughout the observation period, and the mortality for those feeding on non-Bt maize roots was all within 10% (Figure 3A).

On day 7, the corrected mortality of the third to the fifth instar larvae was 54.00–96.60% in the DBN3601T root, 24.67–70.88% in the DBN9501 root, and 6.67–53.31% in the DBN9936 root. The corrected mortality of the third to the fifth instar larvae feeding on Bt maize roots ranked as DBN3601T > DBN9501 > DBN9936. The corrected mortality of different instars feeding on the same Bt maize root was significantly different. The DBN9936 root was significantly more lethal for the third instar than for the fourth and fifth instar larvae, while the lethality of DBN9501 and DBN3601T roots for the third and fourth instar larvae showed no difference but was significantly higher than that for the fifth instar larvae. The higher the instar, the lower the mortality (Figure 3B), and the greater the number of survival days (Figure 3C).

### 3.4. Feeding and Ovipositional Preference of A. ypsilon for Bt Maize and Non-Bt Maize

**Figure 4:**
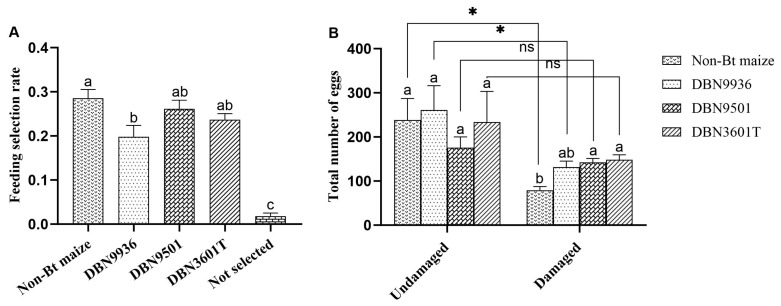
The preference of *A. ypsilon* on different maize. (**A**) The selection rate of neonate larvae to different maize leaves; (**B**) The total number of eggs laid by the moth on the undamaged or slightly damaged Bt maize and damaged non-Bt maize. Different letters indicate significant differences in feeding selection rates and number of eggs between different maize (one-way ANOVA, *p* < 0.05). The symbol * indicates a significant difference in the number of eggs between damaged non-Bt maize and undamaged or slightly damaged Bt maize; ns means no significance (two-sample *t*-test, *p* < 0.05).

The feeding selection rate of newly hatched larvae for non-Bt maize was significantly higher than that for DBN9936 but showed no significant difference from the selection rates for DBN9501 and DBN3601T (Figure 4A).

There were no significant differences in the number of eggs laid on undamaged maize, no difference in the number of eggs laid on the damaged DBN9501 and DBN3601T, but significantly higher than that on non-Bt maize (Figure 4B). This result indicates that moths cannot recognize Bt maize or non-Bt maize when the maize is undamaged. There was a significant difference in the number of eggs laid on non-Bt maize and DBN9936 before and after damage, while there was no significant difference in the number of eggs laid on the DBN9501 maize and DBN3601T maize before and after damage (Figure 4B). This may be because of the degree of damage to non-Bt maize and DBN9936 maize was more serious than that to the two kinds of Bt maize. Therefore, the moth prefers to lay eggs on undamaged or slightly damaged Bt maize.

## 4. Discussion

In this experiment, the Bt protein content in the leaves was consistent with previous experimental results [25,32]. Bt protein was expressed in leaves, stems, and roots; thus, Bt maize could control the whole larval stage of *A. ypsilon*. The content of Bt protein in roots was the lowest, so the key control stage of larvae was before the third instar.

Different target pests have different susceptibilities to Bt maize. For example, the sensitivity of the European corn borer worm to Cry1Ab is much higher than that of *A. ypsilon* [34], and the sensitivity of the fall armyworm to Vip3Aa is much higher than that of the cotton bollworm. Cry3 proteins have good insecticidal toxicity toward western corn rootworms but almost no toxicity toward lepidopteran pests [35]. Currently, the Bt maize approved in China mainly expresses Cry1Ab and Vip3Aa. Therefore, it is of great significance to study the sensitivity of *A. ypsilon* to the insecticidal proteins expressed in Bt maize for the control of various pests. The results of this experiment show that *A. ypsilon* was more sensitive to Vip3Aa and less sensitive to Cry1Ab and Cry1Ab+Vip3Aa, indicating that Bt maize expressing the Vip3Aa protein could effectively control *A. ypsilon*, and the Vip3Aa toxin is more effective in controlling *A. ypsilon* than Cry1Ab [21]. This is consistent with previous research results [36]. However, the expression of Cry1Ab in bivalent Bt maize in this study was much higher than that of Vip3Aa, and the concentration of total Bt protein was used in the calculation, so the LC_50_ of Vip3Aa for *A. ypsilon* was significantly lower than that of Cry1Ab+Vip3Aa. In plants, the insecticidal effect of pyramid Bt maize was better than that of single-toxin maize, so planting Bt maize expressing the Vip3Aa protein could effectively control *A. ypsilon*.

According to the harmful characteristics of *A. ypsilon* larvae, we studied the insecticidal efficacy of different tissues from three varieties of Bt maize on different instar larvae. The results show that all the Bt maize varieties could effectively reduce the survival days of larvae and cause their death. Among them, DBN9501 and DBN3601T expressing the Vip3Aa protein had better insecticidal efficacy for larvae, significantly higher than that of the DBN9936 maize expressing Cry1Ab, and the three Bt maize seedlings tested had better control efficacy for lower-instar larvae than higher-instar larvae. This was related to the higher content of insecticidal protein expressed in Bt maize leaves, along with the lower content of insecticidal protein expressed in the roots, and the older larvae fed on the maize roots with the lowest expression of the Bt protein, which affected the insecticidal efficacy of Bt maize. In production, we should control the larvae with Bt maize before the third instar, to avoid the older larvae undergoing long-term selection from low levels of Bt toxin in the roots that would lead to the generation of resistance.

Insects’ recognition of host plants is important for their growth, development, and reproduction. Research on the interaction between target pests and plants can help in developing appropriate strategies for effective pest control. Studies have shown that plants affected by phytophagous insects produce volatiles that directly affect their feeding and ovipositing behaviors. Insects can identify volatiles or certain components of host plants and, thus, have different feeding and ovipositing choices [37]. Bt crops can induce antifeedant behavior in the cotton bollworm [38]; similar results were found in *Pseudaletia separata* Walker, which shows strong antifeedant activity against Bt maize [39]. This was also confirmed by our research results. The newly hatched larvae exhibited antifeedant behavior toward DBN9936, which may be caused by the fact that the small larvae are highly sensitive to the Cry1Ab protein and are prone to acute gastric toxicity. Many pests would also stop feeding after eating toxin-containing crops. Such avoidance behavior could reduce the intake of toxins. Preventing or reducing the consumption of transgenic insect-resistant crops by this target pest could reduce the damage to maize and, thus, increase its yield. However, it will also reduce the insecticidal efficacy of Bt maize.

The oviposition preference of moths between Bt maize and non-Bt maize was also measured. The results show that moths had no obvious oviposition preference between undamaged Bt maize and non-Bt maize, indicating that moths could not distinguish whether maize expressed the Bt toxin. This is similar to the results of research on the oviposition preferences for Bt crops of various pests [40,41,42,43]. In the field, non-Bt maize is affected by a variety of pests, while Bt corn is less or not affected; thus, we simulated the ovipositional selectivity of moths for both damaged and undamaged corn. The results show that the number of eggs laid on non-Bt maize decreased significantly after damage, while there was no difference in the number of eggs laid on Bt maize before and after damage, indicating that the moth preferred to lay eggs on undamaged or slightly damaged Bt maize. This is consistent with previous studies. Phytophagous insects can identify crop damage by the volatile components of crops and choose to lay eggs on healthier plants. There is significantly less feeding and laying of eggs on damaged plants [44]. Phytophagous insects can not only detect the volatile components of host plants but also identify plants that have been labeled by the chemicals released by the eggs of the same species, thus avoiding damaged plants and choosing to lay eggs in healthy, undamaged plants [45]. Therefore, Bt crops can be used as trap crops for target pests, thereby protecting neighboring non-Bt crops from damage [42], providing new possibilities for the application of Bt crops in integrated pest management.

Planting Bt maize is a highly specific and efficient pest control method. Bt maize, expressing Cry1Ab and Vip3Aa has high control efficacy for a variety of target pests and good control efficacy for *A. ypsilon*, thereby realizing the regional suppression of a variety of pests [46]. Planting Bt maize not only reduces the degree of damage to corn but also reduces the degree of damage to non-Bt host plants [16,17,47]. Bt maize reduces the use of chemical insecticides and can partially replace broad-spectrum insecticides, with obvious ecological benefits [48,49] and no direct toxicity to non-target organisms [50], protecting natural enemy insects and strengthening the impact of biological control in the agricultural ecosystem of maize. The cultivation of Bt maize will promote the biological control function of the entire agroecosystem and increase food production in the long term. A variety of target pests prefer to lay eggs on unharmed Bt maize, which can be used as a trap plant to kill pests and protect other non-Bt crops from damage [51]. An increase in secondary pest populations and the evolution of resistant target pests are potential risks to the sustained control efficacy of Bt maize, and appropriate resistance management and monitoring are required [52]. Because of its high efficiency, Bt maize, which targets a variety of lepidopteran pests, can also support the control of *A. ypsilon* and partially replace the use of large-scale insecticides. In the context of integrated pest management (IPM), Bt maize could effectively combat major and secondary pests by strengthening biological control functions in the Bt maize ecosystem and be combined with other prevention or management measures. In short, planting Bt maize could bring about extremely significant economic and ecological benefits.

## 5. Conclusions

The contents of Bt protein in the leaves and stems of Bt maize events were significantly higher than the content in the roots. The susceptibilities of *A. ypsilon* larvae to the Bt proteins expressed in Bt maize were different, which was the most sensitive to the Vip3Aa protein expressed in DBN9501. The corrected mortality of larvae feeding on DBN9501 and DBN3601T leaves showed no significant difference but was significantly higher than that of those on DBN9936. The insecticidal efficacy of Bt maize roots on third-to-fifth instar larvae ranked as DBN3601T > DBN9501 > DBN9936. Therefore, planting pyramid Bt maize expressing the Vip3Aa protein could effectively control the whole larval stage. The newly hatched larvae had the highest selection rate for non-Bt maize and showed a certain antifeedant behavior toward Bt maize. Moths prefer to lay eggs on undamaged or slightly damaged Bt maize. This study provides a theoretical basis for using Bt maize as a measure to manage *A. ypsilon*.

## Figures and Tables

**Table 1:** Lethal dose of insecticidal protein expressed by Bt maize for *A. ypsilon* larvae.

Maize	Protein	LC_50_ (95% FL)/μg·g^−1^	Slope ± SE	*χ* ^2^	*df*	*p*
DBN9936	Cry1Ab	3.44 (2.16–4.55) a	2.14 ± 1.15	15.31	21	0.81
DBN9501	Vip3Aa	0.82 (0.60–1.02) b	2.53 ± 0.22	8.57	21	0.99
DBN3601T	CrylAb+Vip3Aa	3.39 (2.29–4.21) a	3.31 ±1.76	7.82	21	0.99

95% FL: 95% fiducial limits. Different lowercase letters indicate significant differences in the LC_50_ of the insecticidal protein expressed by different Bt maize seedlings. The significance of a difference was considered according to whether the 95% FL had overlap. SE: standard error. *χ*^2^: Chi-square. *df*: Degrees of freedom. *p* > 0.05 means that the probability model fits well.

## Data Availability

The data were presented in the study.
